# The Flexible Role of Social Experience in the Processing of Abstract Concepts

**DOI:** 10.3390/bs15020190

**Published:** 2025-02-11

**Authors:** Zhao Yao, Yu Chai, Xiaoli He

**Affiliations:** 1 School of Foreign Studies, Xi’an Jiaotong University, Xi’an 710049, China; chaiyu@stu.xjtu.edu.cn; 2 College of Teacher Education, Ningxia University, Ningxia 750021, China

**Keywords:** abstract concepts, socialness, emotional valence, task demands, semantic processing

## Abstract

Multiple representation theories propose that social experience plays an important role in grounding abstract concepts. However, it is less clear how social experience influences the processing of abstract concepts, especially whether this influence is modulated by emotional experience and task demands. To address this question, we orthogonally manipulated the socialness (high vs. low) and emotional valence (positive vs. negative vs. neutral) of abstract words in a lexical decision task (LDT, Experiment 1) and an emotional Stroop task (Experiment 2). Results show that the role of socialness in abstract concept processing was modulated by the concept’s emotional valence, with different patterns between the two tasks. Specifically, positive high-socialness (HS) words elicited slower responses than positive low-socialness (LS) words in the emotional Stroop task, but no such difference was observed in the LDT. In both tasks, however, faster responses were found for negative HS than for negative LS words, and no response differences were observed for neutral HS and LS words. These results provide behavioral evidence for the importance of social experience in the processing of abstract concepts and suggest that concept knowledge derived from social experiences interacts with concept knowledge derived from emotional experiences during lexical–semantic processing. This finding clarifies the heterogeneity of abstract concepts, with positive and negative social words constituting distinct subcategories, and confirms experience-based abstract concepts are inherently flexible, selectively combining with other associated embodied experiences based on task demands, thereby dynamically influencing abstract concept processing.

## 1. Introduction

The referents of abstract concepts are usually not bounded, perceptible physical entities in the external world. Thus, they cannot be directly experienced through the senses (Barsalou, 2003; Borghi et al., 2017). For example, we cannot see or touch “*justice*” or “*friendship*”, but we can understand their meanings, which poses a challenge for strongly embodied theories of concept representation that assume a primary role for sensorimotor information (Pexman et al., 2023). The meanings of abstract concepts, by definition, cannot be learned or experienced through sensorimotor systems. To explain knowledge of abstract concepts, multiple representation views have been proposed, which assume that abstract concepts could be rooted in diverse experiential information, including sensorimotor, linguistic, emotional, and social experiences (Borghi et al., 2017, 2019; Pecher, 2018).

Within this framework, the Words As social Tools (WAT) view explicitly emphasizes the importance of social experience for the grounding of abstract concepts, proposing that social experience is a constitutive semantic dimension of abstract concepts (Borghi et al., 2019). Several semantic feature ratings (Diveica et al., 2023; Villani et al., 2019), property generation tasks (Wiemer-Hastings & Xu, 2005; Recchia & Jones, 2012), behavioral (Diveica et al., 2024b) and neuropsychological (Roversi et al., 2013; Wang et al., 2019) studies have found that social semantic content helps characterize and distinguish subcategories of abstract concepts (e.g., Villani et al., 2019), and affects lexical–semantic processing under different task demands (e.g., Muraki et al., 2022). However, there is limited empirical research on how social experience affects behavioral performance in the processing of abstract concepts and whether this effect is modulated by other relevant embodied experiences, such as emotional experience, across task demands.

In fact, there has been a growing interest in the role that social experience plays in the representation of abstract concepts. On the one hand, some studies have clarified that the socialness of abstract concepts is “the extent to which an abstract word has social relevance by describing or referring to a social characteristic of a person or group of people (e.g., *trustworthy*), a social behavior or interaction (e.g., *fighting*), a social role (e.g., *teacher*), a social space (e.g., *pub*), a social institution (e.g., *hospital*) or system (e.g., *nation*), a social value (e.g., *righteousness*) or ideology (e.g., *feminism*), or any other socially relevant concept” (Pexman et al., 2023; Diveica et al., 2023). Other studies focusing on the issue of heterogeneity in abstract concepts (Mazzuca et al., 2020; Harpaintner et al., 2018; Desai et al., 2018; Troche et al., 2017; Muraki et al., 2022) have reached a relative consensus that abstract concepts can be quite different from each other in terms of the dominant semantic features they activate (e.g., numerical, affective, social and moral). Some fMRI studies have also found that different subcategories of abstract concepts are associated with dissociable brain areas (e.g., Della Rosa et al., 2018; Vargas & Just, 2020; also see Conca et al., 2021, for a review). These studies show that social experience is a key embodied dimension for grounding and distinguishing social abstract concepts from other abstract concepts.

On the other hand, recent empirical work in the field of cognitive science has provided initial evidence for the relationship between social experience and abstract word processing. Some studies using different cognitive tasks have confirmed that the understanding of abstract concepts involves substantial social features. For example, by asking participants to communicate the meaning of words to a partner without using the words themselves, Zdrazilova et al. (2018) found that participants’ speech referred to more people-related and introspective information when communicating the meaning of abstract words. Fini et al. (2021) employed concept-guessing tasks with human–avatar motor interaction and reported that participants needed more cues from partners in order to guess abstract concepts. Diveica et al. (2023) collected socialness norms for over 8000 English words and found that the increased socialness of word meaning was associated with its higher abstractness. In short, socialness is positively correlated with the understanding of abstract words.

Recently, an empirical work by Diveica et al. (2024b) provided preliminary evidence for the facilitated effect of social experience on word processing. The study manipulated word socialness (social vs. nonsocial) and part of speech (nouns vs. verbs), finding that social words were responded to more rapidly and accurately than nonsocial words in a go/no-go noun judgment task. Similarly, Sidhu and Pexman (2016) found that verbs associated with higher bodily experience were remembered more easily and more accurately compared with verbs less associated with bodily experience. Muraki et al. (2022) reported that mental state abstract verbs were processed more quickly than nonembodied abstract verbs in the syntactic classification task. These findings not only corroborate the prediction of multiple representation theories (e.g., Borghi et al., 2019; Barsalou, 2020) that socialness could contribute to word meaning but also align with the semantic richness literature (c.f. Pexman et al., 2002; Pexman, 2012), implying that the richer the semantic content of a word, the better its behavioral performance in word processing.

Despite the initial progress made in exploring the importance of social experience in grounding concepts as a novel experience-based semantic dimension, additional studies are needed to explore how social experience influences behavioral responses to abstract word processing and whether this influence depends on other related embodied experiences such as emotional information (i.e., valence) and varies with ongoing task demands.

A number of studies have shown the close relationship between social and emotional experiences in the understanding of abstract concepts (e.g., Troche et al., 2014, 2017). As noted by Giffard et al. (2015), concepts that describe social behavior (e.g., *polite*, *stingy*) are abstract and generally carry emotional connotations. In other words, processing socially related abstract words may activate the corresponding emotional experience, which we may then consciously or unconsciously feel. For instance, when we consider the word *friendship*, individuals who recall their good friends often experience *joy* and *love*. A large-scale study on socialness rating suggests that abstract words with higher socialness tend to contain more emotional information (Diveica et al., 2023). Neuroimaging evidence indicates that both socialness and emotional valence are processed in the left anterior temporal lobe (ATL) (Dreyer et al., 2015; Binney et al., 2016), but these processes occur in subregions that are partially dissociable (Wang et al., 2019) with different degrees of activation (Mellem et al., 2016). In brief, these studies have demonstrated that while social and emotional experiences are distinct experience-based dimensions, they may interactively contribute to the processing of abstract concepts, due to their modest correlation and the common neural substrates in the ATL.

Compared with the relatively smaller number of empirical works that directly examine the role of social experience in abstract concept processing, several studies have examined the influence of emotional experience (i.e., valence) on abstract word processing. Yet, the results remain inconsistent (e.g., B. Yao et al., 2018; also see Borghi et al., 2017, for a review). Several studies have shown that abstract words with emotional valence (positive and negative) are acquired earlier (Ponari et al., 2018) and are associated with faster and more accurate semantic categorization responses (i.e., decide whether each word referred to a concrete/abstract word) than neutral words (Newcombe et al., 2012; Moffat et al., 2015). In contrast, the facilitating effect of emotional valence over neutral abstract ones is not observed in semantic judgment tasks (i.e., judge whether the probe and target words pair are semantically related) (Wang et al., 2019), or emotionally valenced abstract words are responded to more slowly than neutral ones in the lexical decision task (LDT) (Palazova et al., 2013). The mixed results may be due to the fact that previous studies have rarely considered the heterogeneity of abstract concepts, or rather, different criteria were used to select abstract concepts in previous studies. More specifically, the oversight of the social semantic dimension has resulted in the application of disparate criteria for the selection of abstract concepts in prior research. Research categorizing abstract concepts has shown that emotional and social abstract concepts are two distinct categories (Villani et al., 2019; Diveica et al., 2024a). Specifically, emotional concepts primarily rely on interoceptive signals (sensations originating within the body such as heart beat), as found by Diveica et al. (2024a), and mainly refer to positive (e.g., *happiness*) or negative (e.g., *sadness*) affective states. In contrast, social concepts are characterized by both inner and external grounding, being closely related to exteroceptive signals (sensations originating outside the body such as auditory) with their connotations driven by positive (e.g., *fashion*) or negative social situations (e.g., *separation*). This implies that abstract concepts should be regarded as different types in lexical–semantic processing, given the varying proportions of social and emotional experiences involved. Consequently, socialness and emotionality emerge as separate and identifiable constructs within the semantic space.

Additionally, prior research has suggested that the recruitment of multiple embodied experiences in conceptual representation is flexible and dynamic (for an overview, see Yee & Thompson-Schill, 2016; Khanna & Cortese, 2021; Muraki et al., 2022). This allows experience-based information to be selectively attended to according to task demands, thereby differentially influencing word processing. For example, Connell and Lynott (2014) found that tasks implicitly focusing on visual information in word meaning, like visual lexical decision tasks, facilitate access to words associated with visually experienced concepts (e.g., “*cloudy*” is more often visually associated with than “*noisy*”). Conversely, tasks that implicitly emphasize auditory information, such as reading aloud tasks, facilitate to access words associated with auditorily experienced concepts (e.g., “*noisy*” is more auditorily associated than “*cloudy*”). Frau et al. (2025) investigated the influence of bodily related experience on verb processing across several tasks with different semantic loading. They found that this influence occurs in lexical decisions and memory recognition but not in grammatical decisions.

The task-dependent modulation of experience-based dimensions in word processing is attributable to the degree of activation of semantic properties (e.g., Harpaintner et al., 2020; B. Yao et al., 2022). In tasks that typically require more superficial processing, such as lexical decisions, the primary reliance is on orthographic and lexical properties (Yap et al., 2015). In contrast, tasks involving deeper semantic processing, such as semantic decisions, elicit a more substantial activation of semantic properties (Pexman et al., 2017; also see Frau et al., 2025). However, less is known about how experience-based information during word processing is modulated in tasks that inhibit or interfere with the activation of semantic properties. For example, in the emotional Stroop task, participants are asked to name the ink color of valenced/neutral words while ignoring the semantic content of the words. In this task, the activation of semantic properties may be attenuated or inhibited when a color naming response is required (Williams et al., 1996).

More importantly, several empirical studies have reported that the contribution of experience-based information to word processing differs in tasks with or without semantic interference. For instance, Crossfield and Damian (2021) directly compared behavioral performance on the LDT and on the emotional Stroop task, finding a significant processing advantage for positive words over negative and neutral words in the LDT, whereas valence alone did not produce any effects in the emotional Stroop task. Siakaluk et al. (2014) examined the effects of emotional experience on abstract words in the Stroop task and reported a slower color-naming performance for words with higher emotional experience ratings compared with words with lower ratings. In addition, Arioli et al. (2021) used the modified emotional Stroop task to assess the interactive effect of social content (social vs. nonsocial) and emotion valence (positive vs. negative) on word processing. They found that social context accelerates the processing of positive words but decelerates the processing of negative words. These findings indicate a flexible combination of social and emotional dimensions that influences word processing across varying task demands. Specifically, drawing on studies that exhibit the general processing advantage of positive words in an LTD (for a recent review, see Ferré et al., 2024) and that negative words may be more pronounced in an emotional Stroop task (e.g., Larsen et al., 2006), we posit that the impact of emotional valence on socialness differs across the LTD and emotional Stroop task.

Taken together, there is no dispute that social experience is a key experience-based semantic dimension for grounding and understanding abstract concepts. However, less is known about the influence of social experience on behavioral responses to abstract word processing across varying task demands, especially how this effect is modulated by other relevant embodied experiences, i.e., emotional experience. To this end, the present study conducted two experiment tasks (with or without semantic interference) and employed an identical set of abstract words that differed in socialness (high vs. low) and emotional valence (positive vs. negative vs. neutral) and were matched on several key psycholinguistic variables. Note that social abstract words (hereinafter referred to as social words) used in the present study are characterized by both inner affective states and external social situations, rather than emotional abstract words (i.e., emotional label words), which are primarily ground by internal states (see Villani et al., 2019).

In Experiment 1, participants performed a “word or nonword” LDT that requires attention to the lexicality of the stimulus. In the LDT with lower semantic demands, all semantic properties of words are moderately processed in the same way (answer “yes”), without additional influence from semantic interference. The differences in performance among social words will be due to the contribution of social and emotional content to these words. In Experiment 2, participants performed an emotional Stroop task, which required them to name the ink color of social words. This task was designed to divert attention away from semantic attributes, thereby investigating the impact of social and emotional experiences on the processing of social words, specifically in a context characterized by minimal semantic demands and the concurrent engagement of semantic inhibition. The emotional Stroop task is an adaptation of the classic Stroop task (Stroop, 1935). In the classic Stroop task, participants are presented with colored words (e.g., *red*) printed in congruent (e.g., red) or incongruent (e.g., blue) font colors and are asked to name the font color. Naming latencies are typically longer for incongruent than for congruent words due to interference between the semantic content of the word and task-relevant information, i.e., the color of the word. Accordingly, the semantic interference in the emotional Stroop task is attributed to a highly implicit attentional capture by the semantic content of the word, with poorer performance for a particular type of stimuli with more salient semantic features.

The two tasks were chosen because they allowed us to test the experimental context sensitivity of social and emotional dimensions in abstract word processing by comparing their relative contributions in the LDT, where a *yes* response indicates interference-free semantic processing occurs, and in the emotional Stroop task, in which semantic processing may be attenuated or inhibited when a color naming response is made. That is, differences in behavioral performance to social words may be due to their social and/or emotional content and may also be modulated by additional interference from inhibitory control.

In summary, according to current embodied perspectives that underscore the significance of social experiences in grounding abstract concepts (e.g., Pexman et al., 2023; Borghi et al., 2019), the literature on semantic richness (c.f. Pexman et al., 2002; Pexman, 2012), and the task dependency in word processing (e.g., Siakaluk et al., 2014; Harpaintner et al., 2020; B. Yao et al., 2022), we first hypothesized that behavioral responses to abstract words with high socialness ratings (HS, e.g., *responsibility, rumor*) would differ from those with low socialness ratings (LS, e.g., *direction*, *pain*) in both tasks. That is, the knowledge derived from social experience is automatically activated during the lexical–semantic processing, regardless of task demands. We then assumed that the effect of social experience on abstract word processing would be modulated by emotional experience (i.e., valence), with different patterns of behavioral responses between the two tasks. Based on the limited evidence on the effect of socialness on emotional words (Wang et al., 2019; Arioli et al., 2021), we expected that the processing of HS abstract words might more easily benefit from positive information compared with LS words in the LDT, whereas in the emotional Stroop task, the semantic salience of abstract words might be enhanced by their social and emotional experience, resulting in an amplifying interference effect for HS compared with LS words, especially for negative words.

## 2. Experiment 1—The Role of Socialness in the Processing of Abstract Words with Different Emotional Valence in the LDT

In Experiment 1, we aimed to examine the influence of socialness (high, low) on abstract word processing in the LDT, with moderate semantic demands and without additional semantic interference, and how this influence is modulated by emotional valence (positive, negative, and neutral) of the words.

The stimuli, data, and analysis scripts are available at the Open Science Framework Repository https://osf.io/mwejz (accessed on 8 February 2025).

### 2.1. Method

#### 2.1.1. Participants

As estimated by G*Power 3.1 (Faul et al., 2009), a minimum of 41 participants was required to achieve a 0.25 effect size, 0.05 alpha (2-tailed), and 0.8 power in the multiple regression with socialness and emotional valence as predictors and reaction times as the response variable. Forty-one college students (39 females; 18–22 years old, *M* ± *SD*_age_ = 19.98 ± 2.07 years) participated in Experiment 1. All were native speakers of Mandarin Chinese, right-handed, with normal or corrected-to-normal vision, and without known neurological or mental health issues. Each participant gave informed consent before the study and received monetary compensation. Additionally, the sensitivity analysis (assumed 0.05 alpha (2-tailed), and 0.8 power) with socialness, emotional valence, and their interaction included as predictors revealed the current 41 participants were capable of detecting the smallest effect size for all factors at 0.29 by G*Power, which is considered to be above the medium effect level of 0.25 (Cohen, 1977). This study was approved by the ethics committee of the Health Science Center at Xi’an Jiaotong University (IRB Number: 2022-1534).

#### 2.1.2. Design and Materials

The experiment used a 2 (socialness: high, low) × 3 (emotional valence: positive, negative, neutral) within-participants design. It included 120 abstract words from Wang et al. (2019) and 120 pseudowords that were based on the 120 abstract words and generated by changing one character within different real words. Half of the 120 abstract words contained relatively more social interaction information (e.g., *honor*), while the other half had relatively less social content (e.g., *miracle*). Within each socialness condition, one-third of the words were negatively valenced (e.g., *rumor*, *disease*), one-third were emotionally neutral (e.g., *group*, *syntax*), and one-third were positively valenced (e.g., *honor*, *youth*). All word stimuli are listed in the Appendix on OSF.

Across all six conditions, words were matched for word frequency (obtained from the Beijing Language and Culture University Corpus Center, http://bcc.blcu.edu.cn), age of acquisition (Xu et al., 2021), and character strokes (Chinese Ministry of Culture & State Language Affairs Commission, 1986), as well as concreteness, imageability, and familiarity. Due to the lack of available norms for socialness, valence, arousal, concreteness, imageability, and familiarity in the appendix of Wang et al. (2019), we assessed these six variables through two separate online questionnaires completed by 110 students from two different classes. One class with 74 students (27 males, *M* ± *SD*_age_ = 29.47 ± 11.71 years) was asked to rate the socialness of the words on a 9-point Likert scale (1 = extremely low socialness, 9 = extremely high socialness) according to the norms concluded by Pexman et al. (2023) and Diveica et al. (2023), which index the degree to which words’ referents have social relevance. Another class with 36 students (5 males, *M* ± *SD*_age_ = 20.03 ± 0.81 years) was asked to rate the valence, arousal, concreteness, imaginability, and familiarity of the words on a 9-point Likert scale, with 1 indicating very negative, very calm, highly abstract, difficult to imagine, and unfamiliar, and 9 indicating very positive, very arousing, highly concrete, easy to imagine, and familiar, respectively.

We conducted 2 (socialness: high, low) × 3 (emotional valence: positive, negative, neutral) analyses of variance on all ratings. The socialness ratings of HS words were significantly higher than LS ones (*p*s < 0.001), and a paired test showed positive HS-LS words had a significant difference, and so did negative HS-LS or neutral HS-LS words (*p*s < 0.000). Valence ratings showed significant differences among positive, negative, and neutral words (*p*s < 0.001), with no difference on the positive HS-LS pair nor on negative HS-LS or neutral HS-LS pairs (*p*s ≥ 0.157). Arousal ratings of neutral words were lower than positive and negative ones (*p*s ≥ 0.001), with no difference on positive HS-LS pairs, on negative HS-LS pairs, or on neutral HS-LS pairs (*p*s ≥ 0.503). The mean ratings of these variables (with *SD*s) across conditions are reported in Table 1. It should be noted that the concreteness ratings tend to cluster around the mean (see Supplementary Materials), which may indicate that the words are not strictly abstract. Given that concreteness was negatively correlated with socialness (*r* = −0.32) (Diveica et al., 2023) and positively correlated with familiarity (*r* = 0.54) (Z. Yao et al., 2016), we intentionally selected words with high and low socialness while ensuring high familiarity. This selection criterion contributed to the absence of extreme concreteness ratings in our stimulus set.

#### 2.1.3. Task and Procedure

Participants individually performed the lexical decision task in a behavioral lab. There were eight blocks and 60 trials consisting of 30 words (5 words of each condition) and 30 pseudowords. The order of blocks, and the order of stimuli within blocks, was randomized for each participant. All stimuli were presented in white letters (font: Song typeface, size: 36) over a black background using E-Prime 3.0 (Psychology Software Tools Inc., Sharpsburg, PA, USA) running on a 17-inch monitor (placed at 80 cm from participants’ eyes) with a keyboard.

Each trial began with a fixation cross presented in the center of the screen for 500 ms, followed by the presentation of the stimulus for 2000 ms or until a response was given. The blank screen interstimulus interval (ISI) was 1500–1800 ms. Participants were instructed to respond as quickly and accurately as possible whether each item was a word or a pseudoword by pressing one of two buttons (“Z” or “M” key) placed under their two index fingers. The correspondence between response and keys was counterbalanced across the participants. Before the experiment trials, ten practice trials were presented. The experimental process is shown in Figure 1.

#### 2.1.4. Data Collection and Analysis

To examine differences between HS and LS at each level of valence, Linear Mixed-effects Models (LMMs) were conducted on RT data using the *lme4* (Bates et al., 2015), *lmerTest* (Kuznetsova et al., 2017), and *Emmeans* (Searle et al., 1980) packages in the software R (version 3.6.2). The analyses were carried out on trials of real words with correct responses. According to the criteria for excluding the RT outliers in prior studies (Lund et al., 2019; Sidhu et al., 2020), we excluded trials with RTs greater than 2.5 standard deviations from each participant’s mean (3.28% of the remaining trials). Thus, the analyses reported are based on 9227 experimental observations.

We log-transformed RTs because the raw RT data did not meet the assumption of homogeneity of variance. Then, we fit LMMs on the log-transformed RTs, with socialness (contrast coded: LS = −0.5 and HS = 0.5), emotional valence (dummy coded using neutral words as a reference level), and their interactions as fixed factors. In the initial full model, we included by-subject and by-item varying intercepts and slopes as random factors. Similar to Lund et al. (2019) and Sidhu et al. (2020), we also included Concreteness, Arousal, Familiarity, Imageability, Strokes, (log) Frequency, and Age of Acquisition as control variables (all being scaled before being included in the models). For cases where the full models did not converge, we then fit simpler models that remove the correlations among random effects (Lund et al., 2019) and then employed a stepwise variable elimination approach guided by the Akaike Information Criterion to identify the best-fitting model. Since not all effects are estimated in a single model when a variable contains three or more levels, the models were refitted by separately defining the negative and positive words to serve as the reference level. We report the results of the best-fitting model for RT data: (log) RTs ~ Arousal + Familiarity + Imageability + (log) Frequency + Age of Acquisition + Socialness + Emotional Valence + (1|Subject) + (1|Item) + Socialness: Emotional Valence. Code for this process and each model are available on OSF.

### 2.2. Results

Results reveal a significant facilitating effect of socialness for negative abstract words (*B* = −0.04, *SE* = 0.01, *t* = −3.31, *p* = 0.001), as well as a significant interaction between socialness and negative (vs. positive) valence (*B* = 0.05, *SE* = 0.02, *t* = 2.94, *p* = 0.004) (see Table 2). The follow-up analyses showed that the main effect of emotional valence was not significant (*χ*^2^(2) = 3.22, *p* = 0.200). The main effect of socialness was significant (*χ*^2^(1) = 5.50, *p* = 0.019), and the interaction between socialness and emotional valence was significant (*χ*^2^(2) = 8.85, *p* = 0.012). The simple effect analysis of the interaction revealed the following: at the level of negative valence, the HS words (579 ± 10.6 ms) were responded to significantly faster than LS words (605 ± 10.4 ms) (*t* = 3.17, *p* = 0.002). At the level of positive valence, the difference in RTs of HS (584 ± 10.3 ms) and LS (578 ± 10.4 ms) words did not reach significance (*t* = −0.83, *p* = 0.412). Similarly, at the neutral level, the difference between HS (577 ± 10.4 ms) and LS (593 ± 10.5 ms) words was not significant either (*t* = 1.72, *p* = 0.088) (see Figure 2).

### 2.3. Discussion

The results of Experiment 1 suggest that the role of socialness in abstract word processing is affected by emotional valence in the LDT. Socialness exerts a significant amplifying effect on negative abstract words, showing significantly shorter response latencies for negative HS words than for negative LS words. However, positive HS and LS abstract words showed no significant difference, without a facilitated or inhibited effect of socialness on positive word processing.

The results confirm our prediction that the effect of social experience on abstract word processing is modulated by emotional valence and support the multiple representation views that social and emotional experiences play a joint role in abstract word processing (Pexman et al., 2023; Borghi et al., 2018). However, such findings are inconsistent with our hypotheses, showing that HS abstract words appear to be facilitated by negative rather than positive experiences. That is, the processing of HS abstract words benefits from negative valence. One possible explanation for the advantage of negative HS words could be related to the human evolutionary function of avoiding harmful stimuli from the surroundings, which helps humans increase their chances of survival (Kim et al., 2020; Vaish et al., 2008).

## 3. Experiment 2—The Role of Socialness in the Processing of Abstract Words with Different Emotional Valence in the Emotional Stroop Task

In Experiment 2, we utilized the emotional Stroop task to investigate the impact of socialness (high and low) on the processing of abstract words and to examine how this influence is modulated by the emotional valence (positive, negative, and neutral) of the words. This task requires participants to report the ink color of the presented words, thus inhibiting the activation of semantic properties (i.e., word processing) in favor of a less automatic response (i.e., identifying the color of the word). Longer reaction times (RTs) are indicative of high attentional capture or semantic interference by certain semantic features of the stimuli.

### 3.1. Methods

#### 3.1.1. Participants

Forty-one native Chinese speakers (27 females; 18–22 years old, *M* ± *SD*_age_ = 21.22 ± 1.94) from Xi’an Jiaotong University participated in the experiment for financial compensation. They were right-handed, with normal or corrected-to-normal vision and no history of neurological or psychiatric disorders. All participants gave written informed consent. The experimental procedure was approved by the ethics committee of the Health Science Center at Xi’an Jiaotong University (IRB Number: 2022-1534).

#### 3.1.2. Design and Materials

We used the same 120 abstract words (20 positive/negative/neutral HS, 20 positive/negative/neutral LS) from Experiment 1. For the emotional Stroop task, the standard color palette on Microsoft Word was used to generate prototypical red, yellow, blue, and green versions of each word (font: Song typeface, size: 36), thus resulting in 480 experimental trials. These trials were divided into 8 blocks of 60 trials each and were presented in a different random order generated for each block and participant. We presented every word once per block in a different ink color between blocks; therefore, participants were exposed to all words in all available colors.

#### 3.1.3. Task and Procedure

Testing was conducted in a university laboratory, using the same apparatus as in Experiment 1. In Experiment 2, participants were instructed to categorize the color of the word as quickly and accurately as possible while trying to ignore the word itself. Responses were made by pressing, with the index and middle fingers of both hands, one of four keys on the keyboard (“D”, “F”, “J” or “K” key) corresponding to the left–right spatial locations of four color patches presented below the word (Figure 3). Response–key correspondence was counterbalanced across the participants. Ten practice trials were presented prior to the experiment trials.

Each trial began with a fixation cross presented in the center of the screen for 500 ms, followed by the presentation of the stimulus for 1500 ms or until a response was given. The ISI was 1500–1800 ms. The task procedure is shown in Figure 3.

#### 3.1.4. Data Collection and Analysis

The analysis of LMMs followed those of Experiment 1. After removing incorrect trials, we removed 2.56% of trials with latencies greater than 2.5 standard deviations from each participant’s mean in the emotional Stroop task. Thus, the analyses reported are based on 18,736 experimental observations. The *R* formula of the best-fitting model for RT data is (log) RTs ~ Imageability + Socialness + Emotional valence + (1|Subject) + Socialness: Emotional Valence.

### 3.2. Results

Results reveal significant Stroop interference effects of Socialness for negative LS abstract words (*B* = 0.02, *SE* = 0.01, *t* = 3.50, *p* < 0.001) and for positive HS abstract words (*B* = 0.01, *SE* = 0.01, *t* = 2.54, *p* = 0.011). All the interactions reached significance (*p* ≤ 0.038) (see Table 3). The follow-up analyses showed that the main effects of emotional valence (*χ*^2^(2) = 0.13, *p* = 0.937) and socialness (*χ*^2^(1) = 0.81, *p* = 0.369) were not significant. However, the interaction between socialness and emotional valence was significant (*χ*^2^(2) = 18.21, *p* < 0.001). The simple effects showed that negative HS words (552 ± 10.8 ms) were responded to significantly faster than negative LS words (563 ± 10.8 ms) (*t* = 3.50, *p* < 0.001), whereas positive HS (565 ± 10.8 ms) were responded to significantly more slowly than positive LS (556 ± 10.8 ms) words (*t* = −2.54, *p* = 0.011). The neutral HS (556 ± 10.8 ms) and neutral LS (560 ± 10.9 ms) words showed no significant difference (*t* = 0.54, *p* = 0.588) (see Figure 4).

### 3.3. Discussion

The results of Experiment 2 show that the role of social experience in abstract word processing was influenced by the word’s emotional valence in the emotional Stroop task, in which implicit semantic processing generally was disrupted by color naming. Socialness exerts opposite effects on positive and negative abstract words under the semantic interference condition. That is, longer RTs (i.e., Stroop interference effect) were observed in the implicit processing of positive HS words and negative LS words. Positive HS words were processed more slowly than positive LS words, whereas negative HS words were processed faster than negative LS negative words. The results reveal that greater semantic interference was observed for positive HS words compared with negative ones; conversely, semantic interference was more evident for negative LS words than for positive ones.

These findings are consistent with our hypothesis that there is a joint effect of social and emotional experiences on abstract word processing, with different patterns of behavioral performance between emotional Stroop and LDT tasks. For positive HS words, the combination of social interaction experience and positive emotional feelings could promote “approach” behavioral dispositions, making it more difficult to shift attention from the semantic content to the ongoing task. As a result, the color of positive HS words was named more slowly than positive LS words, showing a stronger interference effect.

Similarly, the survival-related salience of negative HS abstract words was enhanced by their higher social relevance and negative emotional feelings, but such enhancement was likely to elicit “avoidance” behavioral responses, thereby narrowing the attentional scope. According to the attentional spotlight theory (see Fernandes et al., 2011), the focus of attention is constrained around threatening (i.e., extremely negative) stimuli (Derryberry & Reed, 1994; Fenske & Eastwood, 2003). This constraint allows more attentional resources to be allocated for the cognitive task at hand, thereby enhancing performance compared with the processing of nonthreatening stimuli. Consequently, the faster reaction times for negative HS abstract words may reflect that attention is focused on them like a spotlight. This focus leads to the processing of negative HS abstract words being less disrupted by task context interference than negative LS words, resulting in a weaker interference effect.

## 4. General Discussion

The present study investigated the effect of social experience on abstract word processing and whether this effect is modulated by emotional valence and task demands. In Experiment 1, we used the lexical decision task and manipulated two related embodied experiences in the grounding of abstract concepts, socialness (high, low) and emotional valence (positive, negative, and neutral), in order to investigate the influence of emotional experience on the role of socialness in abstract word processing under the task context with lower semantic demands. In Experiment 2, we used the emotional Stroop task, in which word processing can be disrupted or inhibited by the color naming response, to examine whether the effect of socialness on abstract word processing with different emotional valence is modulated by semantic interference.

The results of Experiments 1 and 2 show that the role of socialness in abstract word processing can be flexibly exploited by the different valence of emotional experience, manifesting in different patterns across the two task contexts. First, our findings align with multiple representation views, which propose that sensorimotor and other experience-based (emotion, social, interoception) semantic dimensions, in addition to language experience, contribute to word meaning representation (Borghi et al., 2017, 2019; Pecher, 2018). Moreover, our results provide behavioral evidence supporting the WAT view, which emphasizes the pivotal role of social experience in the formation of abstract concepts (e.g., Borghi et al., 2019). Validating the significance of social experience for abstract concepts elucidates the mechanisms underlying vocabulary acquisition. When examining the developmental trajectory of word meanings, it is observed that abstract words are, on average, acquired later than concrete words (Ponari et al., 2018; Nook et al., 2020). This pattern of vocabulary acquisition can be attributed to the fact that, as children mature, their social interaction experiences increase, thereby enhancing their understanding and acquisition of abstract concepts.

Secondly, the faster response to high-socialness words observed in both experiments, as compared with low-socialness words, aligns with the semantic richness literature (c.f. Pexman et al., 2002; Pexman, 2012), suggesting that the richer the semantic content of a word, the more efficient its processing in behavioral tasks. Moreover, the interaction between social and emotional experiences in abstract word processing corroborates recent rating studies that have identified a connection between social and emotional experiences for grounding abstract concepts (Pexman et al., 2023; Diveica et al., 2023). Notably, this finding also indicates that positive and negative social concepts represent separate subcategories of abstract words. This further suggests that abstract concepts should be categorized into distinct types based on the nature and proportion of their embodied experiences. Finally, our findings corroborate the dynamic view of conceptual representation (Yee & Thompson-Schill, 2016), which posits that the influence of experience-based dimensions on conceptual representation and language processing is variable and modulated by task demands, at least in the context of moderate semantic activation or interference.

Specifically, in the LDT, the enhancing effect of socialness on abstract word processing was observed only for negative valence, as reflected by faster responses to negative HS words than to negative LS words. The processing advantage for negative HS abstract words is consistent with findings from the previous literature suggesting that extremely threatening stimuli have evolutionarily adaptive functions, since it is important for survival to quickly detect, attend to, and avoid negative, aversive stimuli (Kim et al., 2020; Vaish et al., 2008). However, no significant difference in behavioral responses was found between positive HS and LS abstract words, suggesting that the processing of positive abstract words may rely more on their emotional and interoceptive rather than social and exteroceptive experience. Compared with negative social events (e.g., preventing loss of life or limb), positive social experience may be less relevant to survival due to the lower time urgency of dealing with positive social events (e.g., feeding and procreation), although they are of crucial importance in the long term (Pratto & John, 1991). Consequently, positive abstract words benefit little from the social environment, resulting in a similar behavioral performance for positive HS and LS words. An alternative explanation may be that the placement of positive words within semantic space is primarily determined by their valence. In accordance with the informational density hypothesis (Unkelbach et al., 2008), positive information tends to be more densely clustered compared with negative information. As a result, semantic features of concepts exhibit greater similarity within the positive domain. Consequently, the influence of social experience on positive word processing becomes less discernible in tasks that require a moderate level of semantic activation.

In the emotional Stroop task, in which semantic processing of abstract words may be interfered with by color naming, longer response latencies indicate greater attentional interference (Williams et al., 1996). In line with our prediction, the contribution of socialness to valenced abstract words showed different patterns between the two tasks. The results of Experiment 2 show that positive HS words were processed more slowly than positive LS words, whereas negative HS words were processed faster than negative LS negative words. That is, socialness plays an opposite role for positive and negative abstract words in the emotional Stroop task.

For positive HS words, a more pronounced interference effect of socialness on the processing of positive abstract words was observed. A possible explanation is that higher social information combined with positive emotional experience together elicit an *approach* behavioral tendency, which automatically captures and holds attention to the semantic content instead of the ongoing task. That is, the approach motive towards positive HS words leads to a delay in color naming, as the attention is captured by the semantic attributes of the words rather than the task-relevant color features. However, a diminished interference effect of socialness on the processing of negative abstract words was observed. From an evolutionary significance, negative HS abstract words have more survival-related salience than negative LS words. Consequently, these words are more likely to elicit *avoidance* behavioral responses. Such responses narrow the focus of attention, thereby inhibiting word processing and leading to less disruption in color naming tasks. According to the attentional spotlight theory (see Fernandes et al., 2011), the focus of attention is constrained around threatening (i.e., extremely negative) stimuli. In other words, the colors of negative HS abstract words are named more quickly than those of negative LS abstract words, due to the extremely threatening meaning of the former causing more attentional resources to be allocated for the cognitive task at hand, i.e., color naming. Based on the semantic attributes of social abstract words, future research could investigate individual differences in conceptual representation within the context of the emotional Stroop task. For instance, individuals with social phobia may exhibit heightened avoidance of semantic content that aligns with their mental states, such as socially threatening words (e.g., *criticize*), which describe negative evaluations from others. This avoidance reflects a congruence between their mental representation of the word’s meaning and their ongoing experience. Instead, compared with individuals without social phobia, they tend to allocate greater attention to the colors of words. Consequently, this shift in focus can lead to faster responses to the colors of negative HS words, in comparison with other categories of social concepts. Such individual differences in the processing of social words may lend support to the notion that individual experience shapes the semantic system, consistent with the dynamic view of conceptual representation (Yee & Thompson-Schill, 2016).

However, our results are inconsistent with recent empirical work on the possible interaction effect of socialness and emotional valence in the processing of abstract concepts (e.g., Wang et al., 2019; Arioli et al., 2021). Wang et al. (2019) found that socialness did not contribute to the processing of valenced abstract words in the semantic judgment task. Arioli et al. (2021) reported that positive social words are processed faster than positive nonsocial ones, while negative social words are processed more slowly than negative nonsocial ones in the emotional Stroop task. One possible explanation for this inconsistency is the lack of a clearer working definition of socialness. In the study by Arioli et al. (2021), the social connotation of words was categorized into social–emotional words (e.g., *shame*, *fight*) and individual–emotional words (e.g., *pain*, *talent*), including both words that specifically refer to internal feelings and words that are grounded in internal feelings and external situations. By contrast, we used the broader definition based on a large-scale rating study by Diveica et al. (2023), such as social characteristics of people, social behaviors or events, social roles, social institutions, social values, or any other socially relevant concepts. Without agreeing on this definition, at least to some extent, it will be difficult to compare the current evidence on the role of socialness in abstract concepts (Pexman et al., 2023). Based on the aforementioned studies and our empirical findings, we argue for a refined segmentation of social abstract concepts into two pivotal categories: social affective concepts, which are essentially embodied in internal states, and nonsocial affective concepts, which adhere to the broad definition of social concepts. This reclassification is instrumental in interpreting neuroimaging findings, which demonstrate that, despite social and emotional valence-related processing sharing common neural substrates in the left ATL (Dreyer et al., 2015; Binney et al., 2016), they either involve partially separable subregions, with social processing preferentially activating the left anterior superior temporal sulcus and emotional valence processing being more associated with the temporal poles (Wang et al., 2019), or exhibit greater left ATL activation for social affective words as compared with nonsocial affective words (Mellem et al., 2016).

In addition, the varying depth of semantic processing may elucidate the discrepancies observed between our study and the study by Wang et al. (2019). Specifically, in their study with a relatively small sample size, the semantic judgment task was employed, which requires participants to judge the semantic relatedness of probe and target words. This task necessitates the retrieval of semantic information and the comparison of semantic associations and involves alternative responses in terms of the word pairs’ meanings. In contrast, the present study employed the lexical decision task, which induces a moderate semantic activation, and the emotional Stroop task, which does not explicitly require participants to reason about word meanings, entailing the interference of semantic contents. Such discrepancies further underscore that the role of experience-based semantic dimensions in abstract word processing is inherently task-dependent. This finding is consistent with recent research on verb processing (Frau et al., 2025; Muraki et al., 2022) and supports the view that conceptual representations are flexible and vary across task contexts (Yee & Thompson-Schill, 2016).

## 5. Conclusions

In sum, the present study suggests that the role of social experience in the processing of abstract words with varying emotional experiences is relatively flexible and contingent upon task demands. Within a task context characterized by moderate activation of semantic properties, socialness is likely to enhance the salience of negative word processing but not positive words. In a task context of semantic interference, socialness selectively exerts a more pronounced inhibitory effect on the processing of positive HS words and negative LS words. Our findings contribute to a more nuanced understanding of the heterogeneity within social words, highlighting the distinctions between positive and negative social words. More significantly, we provide new evidence for the WAT view, emphasizing the importance of the knowledge derived from social experience for abstract concepts and confirming that experience-based abstract concepts are inherently flexible, selectively combining with other highly associated embodied experiences under different contextual demands.

However, the socialness ratings of negative HS words were lower than those of positive and neutral HS words, and the valence ratings of neutral words were near the positive ones, which may limit the interpretation of the role played by emotional experience in social abstract concept processing and the role played by social experience in neutral abstract concept processing, respectively. Moreover, while the majority of empirical studies have documented the role of experience-based dimensions and the task-related aspects of conceptual representation, these studies have predominantly been limited to Indo-European languages. Future studies should test the cross-linguistic stability of such roles and task dependence or explore how diverse embodied experiences are jointly involved in abstract concept processing across different languages. This approach would thereby provide more comprehensive documentation of cross-linguistic variation for multiple representation accounts.

## Figures and Tables

**Figure 1:**
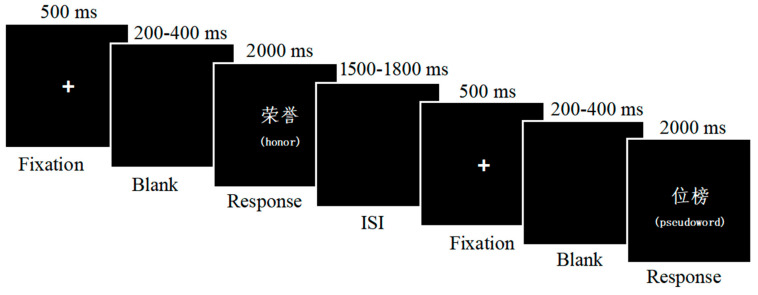
The procedure of the lexical decision task. Notes: The English translations in the parentheses are reported for explanatory purposes, and are not shown during task execution.

**Figure 2:**
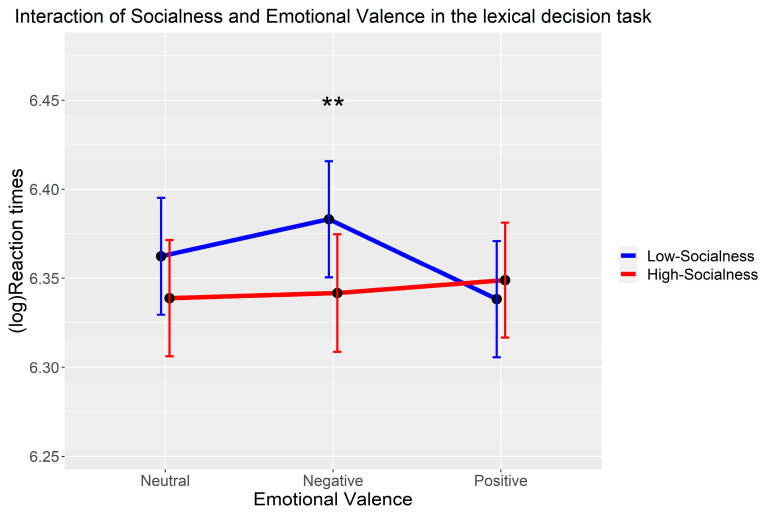
The interaction pattern of socialness and emotional valence in predicting (log) reaction times in the lexical decision task. Notes: ** *p* < 0.01. Err bars indicate 95% confidence intervals.

**Figure 3:**
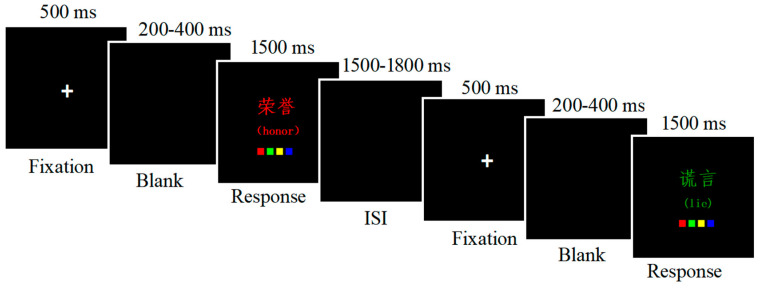
The procedure of the emotional Stroop task. Notes: The left–right spatial locations of four color patches presented below the word remind participants to press one of four keys (“D”, “F”, “J” or “K” key) on the keyboard. The English translations in the parentheses are reported for explanatory purposes, and are not shown during task execution.

**Figure 4:**
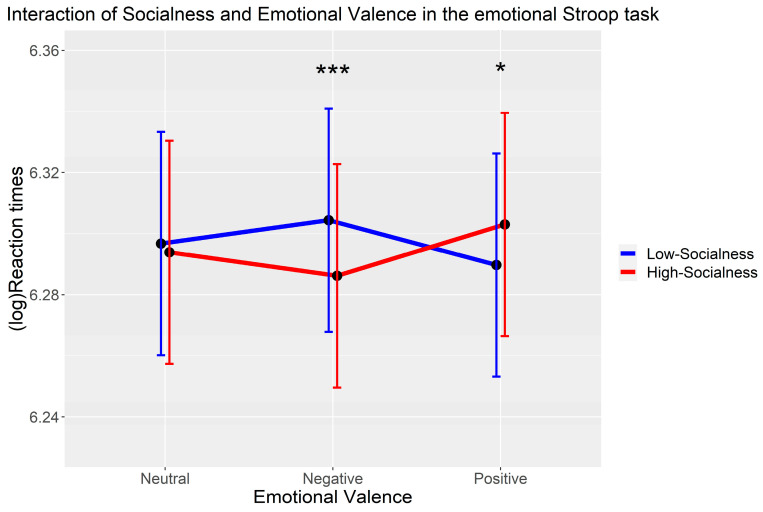
The interaction pattern of socialness and emotional valence in predicting (log) reaction times in the emotional Stroop task. Notes: * *p* < 0.05, *** *p* < 0.001. Err bars indicate 95% confidence intervals.

**Table 1:** The mean ratings of nine variables (with *SD*s) across six conditions.

	High Socialness			Low Socialness		
Variables	Positive	Neutral	Negative	Positive	Neutral	Negative
*N*	20	20	20	20	20	20
Example	荣誉 honor	群体 group	谣言 rumor	青春 youth	句法 syntax	疾病 disease
Socialness	6.29 (0.72)	6.44 (0.56)	5.91 (0.40)	4.73 (0.49)	4.40 (0.32)	4.57 (0.34)
Valence	6.40 (0.31)	5.58 (0.21)	3.95 (0.40)	6.37 (0.40)	5.49 (0.17)	3.96 (0.47)
Concreteness	4.73 (0.54)	4.56 (0.59)	4.67 (0.76)	4.70 (0.64)	4.38 (0.75)	4.42 (0.60)
Arousal	5.84 (0.36)	5.61 (0.24)	5.72 (0.22)	5.89 (0.27)	5.55 (0.26)	5.69 (0.16)
(log) Frequency	4.48 (0.46)	4.64 (0.63)	3.78 (0.57)	4.52 (0.50)	4.60 (0.63)	4.02 (0.67)
Familiarity	7.31 (0.08)	7.25 (0.12)	7.23 (0.08)	7.32 (0.11)	7.31 (0.14)	7.27 (0.13)
Strokes	16.75 (5.77)	15.65 (4.21)	17.30 (3.63)	15.00 (4.16)	16.25 (4.17)	18.30 (5.94)
Imageability	4.36 (0.70)	4.22 (0.53)	4.64 (0.68)	4.16 (0.69)	3.89 (0.56)	4.65 (0.89)
Age of Acquisition	11.23 (1.73)	12.15 (1.12)	11.66 (1.22)	11.61 (2.03)	11.00 (1.23)	11.00 (0.95)

**Table 2:** Linear Mixed-effects Models for predicting (log) reaction times in the lexical decision task.

Fixed Effect	*B*	*SE*	*t*	*p*
Neutral words as the reference level				
Intercept	6.35	0.02	413.31	<0.001 ***
Socialness	−0.02	0.01	−1.80	0.075
Negative	0.01	0.01	1.16	0.248
Positive	−0.01	0.01	−0.73	0.470
Socialness: Negative (neutral vs. negative)	−0.02	0.02	−1.01	0.314
Socialness: Positive (neutral vs. positive)	0.03	0.02	1.88	0.062
Negative words as the reference level				
Intercept	6.36	0.02	409.44	<0.001 ***
Socialness	−0.04	0.01	−3.31	0.001 **
Positive	−0.02	0.01	−1.84	0.068
Socialness: Positive (negative vs. positive)	0.05	0.02	2.94	0.004 **
Positive words as the reference level				
Intercept	6.34	0.02	413.17	<0.001 ***
Socialness	0.01	0.01	0.86	0.390

Notes: *SE* = standard error; observations = 9227; items = 120; subjects = 41. ** *p* < 0.01. *** *p* < 0.001. random effects (*SD*): residuals = 0.17; random intercepts of item = 0.03; random intercepts of subject = 0.09; model’s marginal *R*^2^ = 0.084 and conditional *R*^2^ = 0.308.

**Table 3:** Linear Mixed-effects Models for predicting (log) reaction times in the emotional Stroop task.

Fixed Effect	*B*	*SE*	*t*	*p*
Neutral words as the reference level				
Intercept	6.30	0.02	341.00	<0.001 ***
Socialness	−0.00	0.00	−0.54	0.588
Negative	−0.00	0.00	−0.00	0.998
Positive	0.00	0.00	0.28	0.778
Socialness: Negative (neutral vs. negative)	−0.02	0.00	−2.07	0.038 *
Socialness: Positive (neutral vs. positive)	0.02	0.00	2.19	0.029 *
Negative words as the reference level				
Intercept	6.30	0.02	340.94	<0.001 ***
Socialness	−0.02	0.01	−3.50	<0.001 ***
Positive	0.00	0.00	0.28	0.780
Socialness: Positive (negative vs. positive)	0.03	0.01	4.27	<0.001 ***
Positive words as the reference level				
Intercept	6.30	0.02	341.24	<0.001 ***
Socialness	0.01	0.01	2.54	0.011 *

Notes: *SE* = standard error; observations = 18,736; items = 120; subjects = 41. * *p* < 0.05. *** *p* < 0.001. Random effects (*SD*): residuals = 0.21; random intercepts of subject = 0.12; model’s marginal *R*^2^ = 0.001 and conditional *R*^2^ = 0.240.

## Data Availability

The stimuli, data, and analysis scripts are available at the Open Science Framework Repository https://osf.io/mwejz (accessed on 8 February 2025).

## Supplementary Materials

The following supporting information can be downloaded at: https://www.mdpi.com/article/10.3390/bs15020190/s1. Figure S1: The scatter plot of socialness ratings of stimulus words across six conditions. Figure S2: The scatter plot of emotional valence ratings of stimulus words across six conditions. Figure S3: The scatter plot of concreteness ratings of stimulus words across six conditions. Table S1: The descriptive statistics of socialness ratings of stimulus words across six conditions. Table S2: The descriptive statistics of emotional valence ratings of stimulus words across six conditions. Table S3: The descriptive statistics of concreteness ratings of stimulus words across six conditions. Table S4: Detailed pairwise comparisons in Linear Mixed-effect Models for predicting (log) reaction times in the lexical decision task. Table S5: Detailed pairwise comparisons in Linear Mixed-effect Models for predicting (log) reaction times in the emotional Stroop task.
