# Supplementary material for: The Flexible Role of Social Experience in the Processing of Abstract Concepts
Source: Behav Sci (Basel). 2025 Feb 11;15(2):190. doi: 10.3390/bs15020190 (PMC11851493; doi:10.3390/bs15020190)
Supplement: Supplementary file 1 [file behavsci-15-00190-s001.zip › behavsci-3387416-supplementary.pdf]

Supplementary figures and tables

Figure S1-S3: the scatter plot of the distributions of socialness, emotionality, and concreteness scores across six concept conditions.

Table S1-S3: summarize the minimum, maximum, mean, and standard deviation values of socialness, emotionality, and concreteness scores across six conditions.

Table S4-S5: detailed explanations of the pairwise comparisons in models of two experiments.

Figure S1: The scatter plot of socialness ratings of stimulus words across six conditions

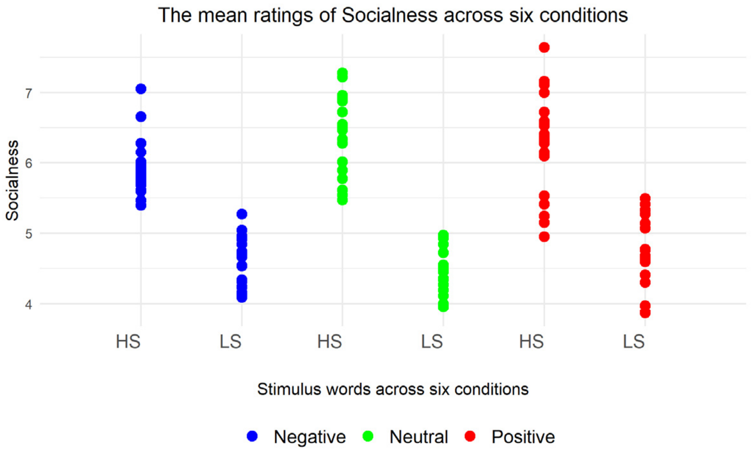

Figure S2: The scatter plot of emotional valence ratings of stimulus words across six conditions

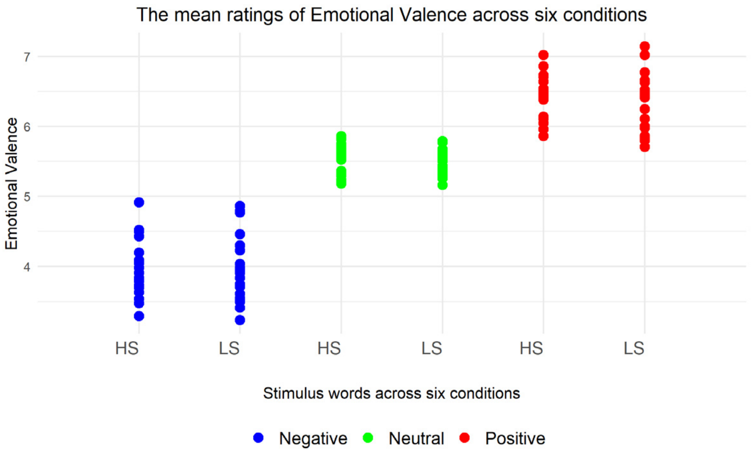

Figure S3: The scatter plot of concreteness ratings of stimulus words across six conditions

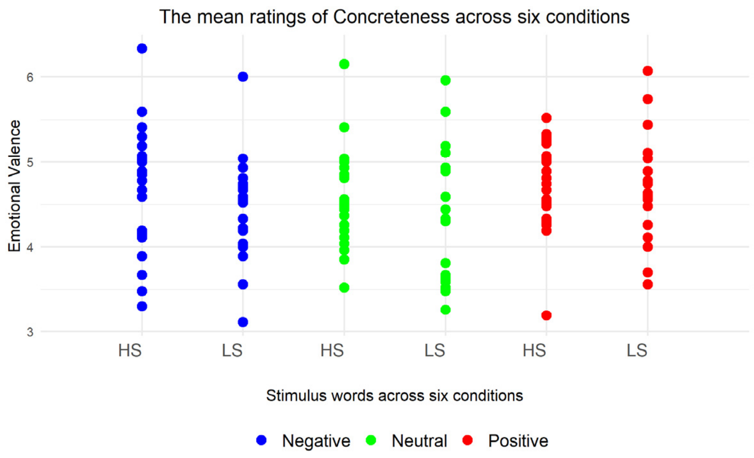

Table S1: The descriptive statistics of socialness ratings of stimulus words across six conditions

	Negative HS	Negative LS	Neutral HS	Neutral LS	Positive HS	Positive LS
Minimum	5.39	4.09	5.47	3.95	4.95	3.86
Maximum	7.05	5.27	7.28	4.97	7.64	5.49
Mean	5.91	4.57	6.44	4.40	6.29	4.73
Standard deviation	0.40	0.34	0.56	0.32	0.72	0.49

Table S2: The descriptive statistics of emotional valence ratings of stimulus words across six conditions

	Negative HS	Negative LS	Neutral HS	Neutral LS	Positive HS	Positive LS
Minimum	3.29	3.23	5.18	5.16	5.86	5.71
Maximum	4.91	4.86	5.86	5.79	7.02	7.14
Mean	3.95	3.96	5.58	5.49	6.40	6.37
Standard deviation	0.40	0.47	0.21	0.17	0.31	0.40

Table S3: The descriptive statistics of concreteness ratings of stimulus words across six conditions

	Negative HS	Negative LS	Neutral HS	Neutral LS	Positive HS	Positive LS
Minimum	3.30	3.11	3.52	3.26	3.19	3.56
Maximum	6.33	6.00	6.15	5.96	5.52	6.07
Mean	4.67	4.42	4.56	4.38	4.73	4.70
Standard deviation	0.76	0.60	0.59	0.75	0.54	0.64

Table S4: Detailed pairwise comparisons in Linear Mixed-effect Models for predicting (log) reaction times in the lexical decision task.

**Fixed Effect**	***B***	***S.E.***	***t***	***p***
Neutral words as the reference level				
Intercept	6.35	0.02	413.31	<.001***
Socialness	-0.02	0.01	-1.80	.075.
Negative	0.01	0.01	1.16	.248
Positive	-0.01	0.01	-0.73	.470
Socialness: Negative	-0.02	0.02	-1.01	.314

Socialness: Positive	0.03	0.02	1.88	.062
Negative words as the reference level				
Intercept	6.36	0.02	409.44	<.001***
Socialness	-0.04	0.01	-3.31	.001**
Neutral	-0.01	0.01	-1.16	.248
Positive	-0.02	0.01	-1.84	.068
Socialness: Neutral	0.02	0.02	1.01	.314
Socialness: Positive	0.05	0.02	2.94	.004**
Positive words as the reference level				
Intercept	6.34	0.02	413.17	<.001***
Socialness	0.01	0.01	0.86	.407
Negative	0.02	0.01	1.84	.068
Neutral	0.01	0.01	0.73	.470
Socialness: Negative	-0.05	0.02	-2.94	.004**
Socialness: Neutral	-0.03	0.02	-1.88	.062

Notes: Observations=9227; Items=120; Subjects=41. * $p < .05$. ** $p < .01$. *** $p < .001$.
Random effects (*SD*): residuals = 0.17; random intercepts of item = 0.03; random intercepts of subject = 0.09; model's marginal $R^2 = .084$ and conditional $R^2 = .308$.

Table S5: Detailed pairwise comparisons in Linear Mixed-effect Models for predicting (log) reaction times in the emotional Stroop task.

**Fixed Effect**	***B***	***S.E.***	***t***	***p***
Neutral words as the reference level				
Intercept	6.30	0.02	341.00	<.001***
Socialness	-0.00	0.00	-0.54	.588
Negative	-0.00	0.00	-0.00	.998
Positive	0.00	0.00	0.28	.778
Socialness: Negative	-0.02	0.00	-2.07	.038*
Socialness: Positive	0.02	0.00	2.19	.029*
Negative words as the reference level				
Intercept	6.30	0.02	340.94	<.001***
Socialness	-0.02	0.01	-3.50	<.001***

Neutral	0.00	0.00	0.00	.998
Positive	0.00	0.00	0.28	.780
Socialness: Neutral	0.02	0.01	2.07	.038*
Socialness: Positive	0.03	0.01	4.27	<.001***
Positive words as the reference level				
Intercept	6.30	0.02	341.24	<.001***
Socialness	0.01	0.01	2.54	.011*
Negative	-0.00	0.00	-0.28	.780
Neutral	-0.00	0.00	-0.28	.778
Socialness: Negative	-0.03	0.01	-4.27	<.001***
Socialness: Neutral	-0.02	0.01	-2.19	.029*

Notes: Observations=18736; Items=120; Subjects=41. * $p < .05$. ** $p < .01$. *** $p < .001$.

Random effects (*SD*): residuals = 0.21; random intercepts of subject = 0.12; model's marginal R^2 = .001 and conditional R^2 = .240.
